# Galectin-4, a Novel Predictor for Lymph Node Metastasis in Lung Adenocarcinoma

**DOI:** 10.1371/journal.pone.0081883

**Published:** 2013-12-10

**Authors:** Takuo Hayashi, Tsuyoshi Saito, Tsutomu Fujimura, Kieko Hara, Kazuya Takamochi, Keiko Mitani, Reiko Mineki, Saiko Kazuno, Shiaki Oh, Takashi Ueno, Kenji Suzuki, Takashi Yao

**Affiliations:** 1 Department of Human Pathology, Juntendo University School of Medicine, Tokyo, Japan; 2 Division of Proteomics and Biomolecular Science, BioMedical Research Center, Juntendo University, Graduate School of Medicine, Tokyo, Japan; 3 Department of General Thoracic Surgery, Juntendo University, School of Medicine, Tokyo, Japan; H. Lee Moffitt Cancer Center & Research Institute, United States of America

## Abstract

Metastasis is still a major issue in cancer, and the discovery of biomarkers predicting metastatic capacity is essential for the development of better therapeutic strategies for treating lung adenocarcinoma. By using a proteomic approach, we aimed to identify novel predictors for lymph node metastasis in lung adenocarcinoma. Two-dimensional sodium dodecyl sulfate polyacrylamide gel electrophoresis showed 6 spots differentially expressed between lymph node metastasis-positive and lymph node metastasis-negative groups in a discovery set. Subsequent mass spectrometry showed that 2 of these spots were derived from galectin-4, and western blot analysis confirmed the overexpression of galectin-4 in metastatic samples. The predictive value of galectin-4 was confirmed by immunohistochemical analysis for a validation set consisting of 707 surgically resected specimens of lung adenocarcinomas (stages I to IV). We observed that 148 lung adenocarcinomas (20.9%) expressed galectin-4, which was significantly associated with variables of disease progression such as tumor size (*p*<0.0001), pleural invasion (*p* = 0.0071), venous invasion (*p* = 0.0178), nodal status (*p* = 0.0007), and TNM stage (*p*<0.0001). By the multivariate analysis, Galectin-4 expression was revealed as one of the independent predictor for lymph node metastasis, together with solid predominant and micropapillary histologic pattern. Furthermore, galectin-4 expression was revealed to be an independent predictor for lymph node metastasis and an adverse survival factor in patients with lung adenocarcinoma of acinar predominant type. Galectin-4 plays an important role in metastatic process of lung adenocarcinoma. Immunohistochemical testing for galectin-4 expression may be useful together with the detection of specific histology to predict the metastatic potential of lung adenocarcinoma.

## Introduction

Lung carcinoma is one of the most common cancers worldwide and the most frequent cause of cancer-related mortality [1]. Driver oncogenic alterations such as *EGFR* mutations and *ALK* fusions have been found in lung adenocarcinoma [2]–[5], which suggests that the treatment outcomes of patients can improve by testing tumors for mutations in the components of specific pathways and applying molecular targeting therapies directed against these components. Despite the advances obtained in treatment modalities by using targeted therapy, metastasis remains an adverse prognostic factor that affects therapeutic approaches for lung adenocarcinoma [6]. To date, numerous molecules such as adenylate kinase-4; annexin A3; caveolin-1; contactin; connective tissue growth factor; corapsin response mediator protein-1; flotillin-1; MUC1; vascular endothelial growth factors A, B, C, and D; and slug have been identified as playing an important role in promoting or inhibiting lung cancer metastasis [7]–[16]. These markers could provide further insights into cancer biology and the processes that lead to cancer metastasis; however, the mechanisms underlying cancer metastasis in lung adenocarcinoma are still poorly understood. Thus, novel biomarkers that can predict metastasis in lung adenocarcinomas need to be developed.

Recent studies have used mRNA-based gene expression profiles to identify molecular signatures that are discriminative of lung adenocarcinoma differentiation and prognosis [17]–[20]. The use of proteomics in cancer research has provided a new approach for identifying differential proteins associated with tumor progression, thereby creating new opportunities to screen novel biomarkers.

Here, we performed a quantitative expression study to identify regional lymph node (LN) metastasis-related biomarkers in the initial stages of metastasis by using two-dimensional sodium dodecyl sulfate polyacrylamide gel electrophoresis (2D SDS-PAGE) and mass spectrometry. We found that galectin-4 was one of the independent predictor for LN metastasis in lung adenocarcinoma. Furthermore, galectin-4 expression in acinar histology may predict adverse clinical outcomes as well as LN metastasis.

## Materials and Methods

### Patients

We screened the archives of the Department of Human Pathology, Juntendo University School of Medicine, from May 2008 to August 2012 for all patients who underwent complete resection of lung adenocarcinomas and obtained their clinicopathological data, including those for gender, age, tumor size, lymphovascular invasion, lymph node and distant metastases, resection type, and adjuvant therapy. All the tumors were resected at the Department of General Thoracic Surgery, Juntendo University Hospital. Diagnoses were made according to the criteria of the current WHO classification of lung carcinomas [21] and the proposal of the International Association for the Study of Lung Cancer (IASLC), American Thoracic Society (ATS), and European Respiratory Society (ERS), which is a novel classification system for lung adenocarcinomas that relies on the tumor histological architecture [22]. Criteria from the seventh edition of the International Union Against Cancer (UICC)/American Joint Committee on Cancer TNM classification were also used for all lung adenocarcinomas. All patients were followed up regularly by physical and blood examinations with mandatory screening X-ray, computed tomography, or magnetic resonance imaging.

### Clinicopathological information

Our archives included data for 797 patients with lung adenocarcinoma. Of these, patients who received preoperative chemotherapy or radiotherapy, or patients whose tissue samples were not available for validation were excluded. We examined the remaining 707 lung adenocarcinomas from 686 patients (stages I to IV). Two board-certificated pathologists (TH and TS) reviewed the histologic features of the lung adenocarcinomas. The lung adenocarcinoma subtypes included 28 invasive mucinous adenocarcinomas, 2 colloid adenocarcinomas, and a well-differentiated fetal adenocarcinoma, as defined by consensus [22]. None of our patients were diagnosed with enteric adenocarcinoma. This study was approved by the Ethics Committee of Juntendo University School of Medicine (2012014). Informed, written consent was obtained from all patients.

### Preparation of lung adenocarcinomas tissues homogenate

We prepared a discovery set consisting of 10 lung adenocarcinomas (5 cases with LN metastasis and 5 cases without LN metastasis) in order to examine the differentially expressed proteomic profiles. All samples of discovery set were prospective accumulation for the purpose of this study. Lymph node dissection was performed at N2 level in all cases with LN metastasis and three of five cases in non-metastasizing group; the exceptions were case N1 and N3 whose nodal dissection was done at N1 level. The clinicopathological features of the set are summarized in Table S1. The frozen lung adenocarcinomas tissues (26∼142 mg) were minced with scissors and homogenized in 250∼500 ul RIPA buffer (Cell Signaling Technology, Inc. Beverly, MA, USA) using the Potter homogenizer set at 20 strokes on ice. Nuclear fractions and debris were removed by centrifugation at 700 x *g* for 10 min at 4°C. We used the supernatants (20 ug) for one or two-dimensional electrophoresis. The signals were analyzed using ImageQuant TL software.

### Two-dimensional sodium dodecyl sulfate polyacrylamide gel electrophoresis

Two-dimensional SDS gel electrophoresis, in situ alkylation, and in gel digestion were performed as described previously [23], [24]. For the first-dimensional electric focusing of the lung adenocarcinomas tissues (20 ug protein from each sample), IPGphor strips (11 cm) pH 3–10 were used (GE Healthcare UK Ltd.). The lung adenocarcinomas tissues were diluted by the electric focusing solution containing 7 M urea, 2 M thio-urea, 4% CHAPS, 65 mM dithioerythritol (DTE), 2% carrier ampholyte at pH 3–10 and bromophenol blue (BPB). Then, electric focusing was performed in the following steps; held at 30 V for 8 h held at 60 V for 8 h, increasing voltage from 60 to 200 V for 1 h 200 to 500 V for 1 h 500 to 1000 V for 1 h 1000 to 8000 V for 1 h and held at 8000 V for 2 h, *i.e.*, a total of 22.3 k Vhr. Before the second-dimensional SDS-PAGE, the strips were immersed in a solution containing 50 mM Tris-HCl, pH 8.5, 6 M urea, 30% glycerol, 2% SDS, 130 mM DTE, and 0.005% BPB for 10 min in order to reduce SH-residues of proteins, then placed onto the SDS-PAGE gels. SDS-PAGE (stacking gel 4% acrylamide, running gel 10% acrylamide) was performed as follows; rerun at 10 mA for 1 h; run at 70 mA for 2.5 h. The proteins on the gels were stained by the Plus One silver stain kit (GE Healthcare UK Ltd.) and measured by the image analyzer. 2D SDS-PAGE images were scanned using a calibrated Bio-Rad GS 800 densitometer and the signals were analyzed using PDQUEST Version 7.1 software (Bio-Rad Laboratories, Inc. CA, USA). In gel digestion were performed as described previously [25], [26]. Comparisons of 2D gels were carried out by hand.

### Mass spectrometric analysis to identify proteins

Peptide mapping was carried out using the Triple TOF 5600 mass spectrometer systems, which consisted of nano-ESI and TOF, (AB SCIEX MA, USA). The Triple TOF 5600 mass spectrometer was combined with Eksigent NanoLC-Ultra system + cHiPLC-nanoflex system (AB SCIEX MA, USA) with attached 75 um id X 15 cm Chrom XP C18-CL column. The solvent system consisted of (A) 0.1% formic acid/2% acetonitrile, and (B) 0.1% formic acid/90% acetonitrile. The solvent program was gradient at 0.95%B/min for 40 min and 90%B for 5 min (gradient: 2%B 40%B (40 min) wash: 90%B (5 min) conditioning: 2%B (20 min)). The flow rate was 300 nl/min. Identification of proteins was performed using Protein Pilot 4.0 software (AB SCIEX MA, USA).

### Western blotting

SDS–PAGE was performed using 10∼20% gradient acrylamide gel. After electrophoresis, the proteins on the gel were transferred onto polyvinylidene difluoride (PVDF) membrane (Immobilon P, 0.45 lm, Millipore, USA). Membrane was incubated overnight at 4°C with 5 ug/ml of the first antibody (goat polyclonal antibodies against galectin-4, sc19286, Santa Cruz Biotechnology, Santa Cruz, California, USA and rabbit polyclonal antibody against PPIB, HPA012720; SIGMA Life Science, MO, USA), followed by secondary anti-goat or anti-rabbit IgG-HRP (5000-fold dilution, Jackson ImmunoResearch Laboratories, Inc. West Grove, PA, USA). The antibody reactivity of galectin-4 was developed by ECL Prime (GE Healthcare UK Ltd.), then detected by ImageQuant LAS 4000 mini (GE Healthcare UK Ltd.). The signals were analyzed using ImageQuant TL Version 7 software.

### Immunohistochemistry

All tissues were fixed in 10% buffered formalin, embedded in paraffin after routine processing. Tissue sections (thickness, 4 um) were deparaffinized and hydrated. Endogenous peroxidase activity was blocked with 1.3% hydrogen peroxide in methanol at room temperature. Immunohistochemical examinations were performed using goat polyclonal antibodies against galectin-4 (sc19286; 1∶100 dilution; Santa Cruz Biotechnology), and rabbit polyclonal antibody against PPIB (HPA012720; 1∶600 dilution; SIGMA Life Science), and 3, 3′-diaminobenzidine tetrahydrochloride as the chromogen. Three pathologists (TH, KH, and TS) reviewed the representative sections stained with the anti-galectin-4 and anti-PPBI antibodies in a blinded manner with regard to the clinical data. Samples for which >10% of tumor cells stained strongly positive with antigalectin-4 antibody were considered to show galectin-4 expression. On the other hand, samples for which >50% of tumor cells stained strongly positive with anti-PPBI antibody were considered to show PPBI expression.

### Statistical analysis

The association between galectin-4 expression and various clinicopathological features was evaluated using the κ^2^ test. The logistic regression model was used to identify independent predictive factors for lymph node metastasis in univariate and multivariate analyses. Overall survival and recurrence-free survival was measured in patients who underwent surgery from 2008 to 2009. The follow-up period lasted up to 213 weeks (mean: 151 weeks). Overall survival was calculated from the time of surgery to the time of cancer-related death. Recurrence-free survival was calculated from the time of surgery to the time of recurrence after surgery or death. Overall survival and recurrence-free survival was obtained by the Kaplan-Meier method, and log-rank testing was used to evaluate the statistical significance of difference. Cox regression analysis was used to evaluate the prognostic significance of clinicopathological factors. p<0.05 was considered statistically significant. All statistical analyses were performed using the statistical program R (http://cran.r-project.org)

## Results

### Proteomic analysis indicated galectin-4 as a candidate biomarker for predicting LN metastasis

We performed 2D SDS-PAGE to compare the protein expression profiles of lung adenocarcinomas with and without LN metastasis and identified 6 spots with different intensities (Figure S1). Subsequent mass spectrometry indicated that 2 spots that showed overexpression in regionally metastasized primary tissues corresponded to galectin-4 (Figure S2) and 1 spot corresponded to peptidyl-prolyl cis-trans isomerase B (PPIB). All the spots identified in the non-metastasizing group corresponded to glyceraldehyde 3-phosphate dehydrogenase (GAPDH). Both Galectin-4, a carbohydrate-binding protein belonging to the galectin family, and PPIB were chosen for further validation. Especially, galectin-4 is a protein belonging to the galectin family which are known to play a role in cancer cell activities [27] and little is known about galectin-4 expression in lung adenocarcinoma.

### Validation of galectin-4 expression by western blot analysis

**Figure 1:**
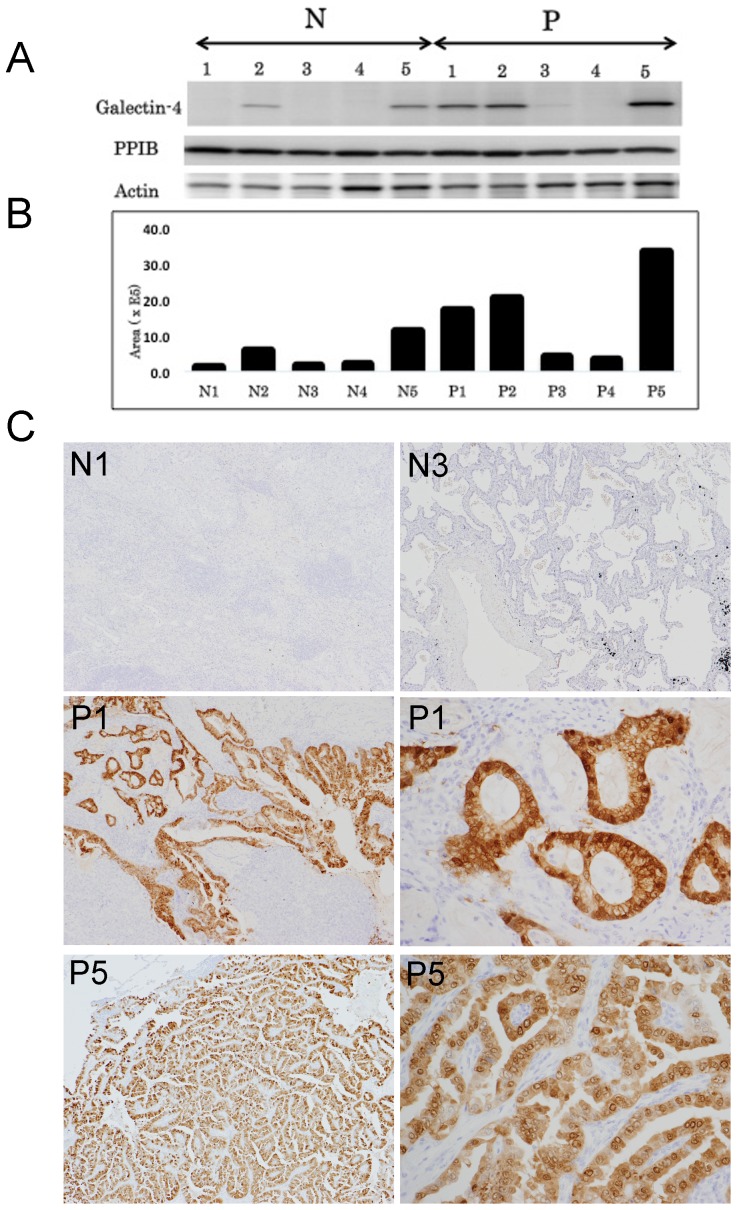
Validation of the differential expression of galectin-4. A. A representative result of western blotting for galectin-4 expression and PPIB, together with β-actin as internal control. Three of 5 lung adenocarcinomas with LN metastasis expressed galectin-4 at higher levels than those without LN metastasis. One lung adenocarcinoma with LN metastasis (P3) showed a slightly increased expression level of galectin-4. PPIB expression was observed almost equally in all samples examined. B. Histogram showing the relative expression levels of galectin-4. C. Immunohistochemical expression patterns of galectin-4 in normal lung tissue and lung adenocarcinoma tissues. Lung adenocarcinomas without lymph node metastasis (N1 and N3) showed that both carcinomas and normal lung tissue did not express galectin-4, whereas lung adenocarcinomas with lymph node metastasis (P1 and P5) showed that galectin-4 was strongly expressed in carcinoma cells (magnification ×20). The high-power view of lung adenocarcinomas with lymph node metastasis showed nuclear, cytoplasmic, and membranous expression of galectin-4 (magnification ×100).

Western blot analysis was performed to confirm the differential expression of galectin-4 and PPIB in 10 discovery samples. Four of the 5 (80%) regionally metastasized primary tissue samples expressed galectin-4, whereas 2 of the 5 (40%) non-metastasized primary tissue samples expressed galectin-4 (Figure 1A and B). Furthermore, 3 samples in the regionally metastasized group expressed galectin-4 at higher levels than the non-metastasized group, whereas only 1 sample (P3) in the metastasized group showed a slightly increased expression level of galectin-4. These results were consistent with the 2D SDS-PAGE results. On the other hand, PPIB expression was observed almost evenly in all 10 examined cases on western blotting (Fig. 1A).

### Immunohistochemical validation showed cytoplasmic, nuclear, and membranous expression of galectin-4 in lung adenocarcinoma

In the discovery samples, the proportion of carcinoma cells that showed strong expression of galectin-4 on immunohistochemical analysis was almost consistent with the expression levels of galectin-4 determined by western blotting. Immunohistochemical analysis showed cytoplasmic, nuclear, and membranous expression of galectin-4 in carcinoma cells; galectin-4 was frequently observed to localize much more frequently to the nucleus and cytoplasm than to the membranes. In addition, only a small number of stromal cells around carcinoma cells expressed galectin-4, whereas no expression was detected in the immune cells surrounding carcinoma cells and normal lung tissues (Figure 1C).

### Relationship between galectin-4 expression in lung adenocarcinomas and clinicopathological factors

We immunohistochemically evaluated the clinicopathological significance of galectin-4 in lung adenocarcinoma. From 707 lung adenocarcinoma samples, 148 adenocarcinomas from 147 patients showed galectin-4 expression (20.9%). Galectin-4 expression was detected in 34 lepidic (17.0%), 60 acinar (28.4%), 21 papillary (27.3%), 9 solid (11.7%) and 1 micropapillary predominant adenocarcinomas (100%). Additionally, specific lung adenocarcinoma subtypes, including 19 invasive mucinous adenocarcinomas (67.9%), and 2 colloid carcinomas (100%), expressed galectin-4. On the other hand, only 1 adenocarcinoma in situ (1.5%) and 1 minimally invasive adenocarcinomas (2.2%) expressed galectin-4. Our study included 21 patients with 2 primary lung adenocarcinoma lesions in the same lobe; both lesions from 1 patient showed galectin-4 expression, whereas none of the adenocarcinomas in the remaining 20 patients showed galectin-4 expression. Next, we analyzed the correlation between galectin-4 expression and various clinicopathological variables including nodal status (Table 1). Galectin-4 expression was found to be associated with tumor size (*p*<0.0001), pleural invasion (*p* = 0.0071), venous invasion (*p* = 0.0178), nodal status (*p* = 0.0007), and TNM stage (*p*<0.0001). These data showed that galectin-4 expression was associated with more aggressive and progressive tumor phenotypes in lung adenocarcinomas. Furthermore, logistic regression analysis showed that galectin-4 expression could independently predict LN metastasis in patients with lung adenocarcinomas (*p* = 0.0021; Table 2). These results provide unique evidence that galectin-4 can be a candidate biomarker for predicting LN metastasis in lung adenocarcinoma. However, galectin-4 showed neither significant correlation with overall nor recurrence-free survival (Figure S3).

**Table 1:** The relationship between clinicopathological variables and Galectin-4 expression and univariate analysis of prognostic factors.

	Galectin-4 positive	Galectin-4 negative	Correlation	OS	RFS survival
	(no. cases)	(no. cases)	χ2 (*p*)	Log-rank (*p*)	Log-rank (*p*)
Age			0.0422	0.464	0.375
<60	27	143			
61<	120	396			
Gender			0.0663	0.98	0.0266
Female	87	273			
Male	60	266			
Size			<0.0001	<0.0001	0.0173
<2 cm	45	316			
2–3 cm	46	132			
3–5 cm	37	83			
5 cm<	20	28			
Pleural invasion			0.0071	0.0117	0.0192
pl0	96	424			
pl1-pl3	52	135			
Nodal status			0.0007	0.0955	<0.0001
N0	105	466			
N1/N2	43	93			
Histology			<0.0001	0.000399	<0.0001
AIS	1	64			
MIA	1	44			
Invasive adenocarcinoma					
Lepidic predominant	34	166			
Acinar predominant	60	151			
Papillary predominant	21	56			
Solid predominant	9	68			
mucinous adenocarcinoma	19	9			
Lymphatic invasion			0.3001	<0.0001	0.0132
Absent	100	402			
Present	48	157			
Venous invasion			0.0178	<0.0001	0.00839
Absent	93	407			
Present	55	152			
Micropapillary pattern (%)			0.1225	0.614	0.798
< or = 5	134	526			
>5	14	33			
TNM stage			<0.0001	<0.0001	0.241
IA or IB	90	422			
IIA or IIB	29	41			
IIIA or IIIB	27	65			
IV	2	11			
Galectin-4				0.647	0.905

OS, Overall survival; RFS, recurrence-free survival; AIS, Adenocarcinoma in situ; MIA, Minimally invasive adenocarcinoma.

**Table 2:** Univariate and multivariate analysis of predictive factors for nodal metastasis.

Variable	OR	95% CI	*p* value
Univariate analysis			
Age (>60 vs. < or = 60)	1.109	0.699 – 1.761	0.6586
Gender (male vs. female)	2.069	1.405 – 3.048	0.0002
Size, cm (>3.0 vs. < or = 3.0)	5.314	3.555 – 7.944	<0.0001
Histology (solid vs. non-solid predominant)	8.903	5.361 – 14.785	<0.0001
Lymphatic invasion (present vs. absent)	10.663	6.960–16.336	<0.0001
Venous invasion (present vs. absent)	11.941	7.742–18.420	<0.0001
Pleural invasion (present vs. absent)	7.532	4.060 – 13.974	<0.0001
Micropapillary pattern,% (>5 vs. < or = 5)	2.052	1.350 – 3.120	0.001
Galectin-4 (positive vs. negative)	2.052	1.350 – 3.120	0.001
Multivariate analysis			
Gender (male vs. female)	0.959	0.578 – 1.591	0.8705
Size, cm (>3.0 vs. < or = 3.0)	2.125	1.285 – 3.514	0.0036
Histology (solid vs. non-solid predominant)	5.514	2.872 – 10.585	<0.0001
Lymphatic invasion (present vs. absent)	2.978	1.529 – 5.800	0.0014
Venous invasion (present vs. absent)	2.345	1.219 – 4.513	0.0105
Pleural invasion (present vs. absent)	1.826	1.085 – 3.073	0.0246
Micropapillary pattern,% (>5 vs. < or = 5)	5.037	2.392 – 10.604	<0.0001
Galectin-4 (positive vs. negative)	2.373	1.377 – 4.089	0.0021

OR, odds ratio; CI, confidence interval.

**Figure 2:**
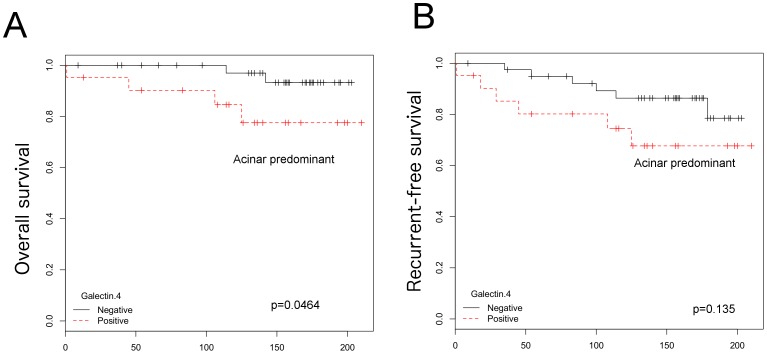
Kaplan-Meier plots for lung adenocarcinoma belonging to acinar predominant type. Galectin-4-negative group has survival advantage compared to galectin-4-positive group (p = 0.0464) (A). The difference in the recurrence-free survival was not significant between both groups (B).

Lastly, we investigated the association of galectin-4 with LN metastasis and survival rate in each histologic subtype. Using the κ^2^ test, galectin-4 expression was found to be associated with the nodal status (*p*<0.0001) in adenocarcinomas of acinar predominant subtype, which is the most common subtype of invasive lung adenocarcinomas (Table 3). Moreover, in acinar predominant subtype, logistic regression analysis showed that galectin-4 expression could independently predict LN metastasis (*p* = 0.00517; Table 4), and overall survival rate was significantly higher in the galectin-4-negative group than in the positive group (*p* = 0.0464) (Figure 2). However, in multivariate Cox regression analysis, no factors including nodal status and galectin-4 remained as the significant independent prognostic factors of decreased overall survival rate (Table 5). Additionally, galectin-4 expression showed neither significant correlation with LN metastasis nor overall and recurrence-free survival in other histological subtypes (Figure S3).

**Table 3:** The relationship between nodal status and Galectin-4 expression in each subtype.

	Galectin-4 positive	Galectin-4 negative	Correlation χ2 (*p*)
	no. cases (%)	no. cases (%)	
All invasive adenocarcinomas			0.02701
N0	103 (22.3)	358 (77.7)	
N1/N2	43 (31.6)	93 (68.4)	
Lepidic predominant			0.3605
N0	34 (17.3)	162 (82.7)	
N1/N2	0 (0.0)	4(100.0)	
Acinar predominant			<0.0001
N0	33 (21.0)	124 (79.0)	
N1/N2	27 (50.0)	27 (50.0)	
Papillary predominant			0.776
N0	12 (26.1)	34 (73.9)	
N1/N2	9 (29.0)	22 (71.0)	
Solid predominant			0.6521
N0	3 (9.7)	28 (90.3)	
N1/N2	6 (13.0)	40 (87.0)	

**Table 4:** Univariate and multivariate analysis of predictive factors for nodal metastasis in acinar predominant type.

Variable	Univariate analysis (*p*)	Multivariate analysis (*p*)	OR	95% CI
Age (>60 vs. < or = 60)	0.71384			
Gender (male vs. female)	0.93567			
Size, cm (>3.0 vs. < or = 3.0)	0.19116			
Lymphatic invasion (present vs. absent)	0.00206	<0.0001	6.629	3.272 – 14.18
Venous invasion (present vs. absent)	0.52355			
Pleural invasion (present vs. absent)	0.13554			
Micropapillary pattern,% (>5 vs. < or = 5)	0.10600			
Galectin-4 (positive vs. negative)	0.00517	0.0011	3.303	1.617 – 6.835

OR, odds ratio; CI, confidence interval.

**Table 5:** Multivariate Cox regression analysis of overall and recurrence-free survivals in acinar predominant type.

Variable	Overall survival		Recurrence-free survival	
	HR (95% CI)	*p*	HR (95% CI)	*p*
Size (>3.0)	1.162 (0.1879–7.191)	0.871	0.8208 (0.2322–2.902)	0.7592
Nodal status (positive)	2.431 (0.4219–14.010)	0.320	5.0311 (1.4544–17.403)	0.0107
Galectin-4 (positive)	3.753 (0.5598–25.163)	0.173	1.3795 (0.3980–4.782)	0.6120

HR, hazard ratio; CI, confidence interval.

### Validation of PPIB expression

Immunohistochemical analysis showed cytoplasmic expression of PPIB in carcinoma cells. In addition, stromal cells or alveolar macrophages expressed PPIB. Of 211 lung adenocarcinomas with acinar predominant type, 89 adenocarcinomas expressed PPBI (42.2%). However, PPIB expression showed no significant correlation with LN metastasis (*p* = 0.4777). Furthermore, overall or recurrence-free survival rate was rather higher in the PPIB positive group than in the negative group, though this was not statistically significant (Figures S4A and B).

## Discussion

Using 2D SDS-PAGE and mass spectrometry, galectin-4 was observed to be differentially expressed in lung adenocarcinomas with and without LN metastasis. Galectins are defined as proteins containing a canonical carbohydrate recognition domain with affinity for β-galactosides [28]–[30]. To date, 14 galectins have been identified, and galectin-4 is encoded by *LGALS4*. Due to growing evidence, the important role of galectins as central regulators of immune and inflammatory responses is indisputable [31]. Nevertheless, the presence of putative binding sites for a number of transcription factors, including c-Rel, a subunit of nuclear factor κB (NF-κB), in the promoter region of *LGALS4* suggests that galectin-4 is a downstream component of the NF-κB pathway [32], which is involved in carcinogenesis [33]. The overexpression of galectin-4 has also been shown in hepatocellular carcinoma and gastric cancer cells with increased metastatic potential at the mRNA level [34], [35]. Furthermore, galectin-4 protein overexpression has been demonstrated in colon adenocarcinoma, and ductal and lobular carcinomas of the breast by using immunohistochemical analysis [32]. Therefore, our results, taken together with those of previous reports, suggest that galectin-4 may have a metastasis-promoting effect. In contrast, the cellular expression of galectin-4 has been reported to be lower in colorectal adenocarcinomas than in normal colon tissues, suggesting that galectin-4 have tumor-suppressive effects in the development and progression of colorectal carcinomas [36].

The present study showed that galectin-4 is localized to the cytoplasm, nucleus, and focal membranes in lung adenocarcinomas. In contrast, breast and colon carcinomas express galectin-4 almost exclusively in the cytoplasm [32]. Galectins are synthesized in the cytoplasm and are subsequently transported into the nucleus, potentially via both active transport and passive diffusion [37]. Galectin-3, which is one of the most studied galectins, is an important regulator of a broad range of cancer cell activities and plays important roles in cancer cell growth, transformation, apotosis, angiogenesis, adhesion, invasion, and metastasis. The divergent effects of galectin-3 on cell activities result from its localization to the following parts of the cell: the cytoplasm, nucleus, and cell surface [27]. Based on the observations, galectin-4 could have divergent function in the development and progression of cancer, depending on its localization in lung adenocarcinoma, compared to that of breast and colon carcinomas.

Our evaluation of immunohistochemical galectin-4 expression clearly showed that galectin-4 expression is associated with clinicopathologic variables of disease progression such as tumor size, pleural or venous invasion, nodal status, and TNM stage. By the multivariate analysis, galectin-4 expression was revealed as an independent predictor with intermediate power for lymph node metastasis, although the odds ratio was lower than those of histological features (solid predominant and micropapillary pattern). Additionally, when we examined statistically in each subtype, galectin-4 expression was significantly associated with the nodal status and poor survival in the acinar predominant subtype. Recent evaluation of the new IASLC/ATS/ERS classification system demonstrates that sub-classifications have predictive value in themselves [38]. Thus patients with acinar predominant subtype can be further divided into two groups: the galectin-4-positive group and the galectin-4-negative group, the farmer having a worse prognosis, although long-term follow-up is needed to confirm this observation.

It is unclear how galectin-4 is involved in the acquisition of metastatic potential in lung adenocarcinoma cells. Galectin-4 expression was associated with the presence of venous invasion, but not with the presence of lymphatic invasion, speculation follows that Galectin-4 may develop metastasis of lung adenocarcinoma cells via venous invasion. In addition, galectins are known to be secreted through a nonclassical pathway [39]. Recently, the levels of serum galectins, including that of galectin-4, were reported to increase in patients with colon and breast cancer [40]. Other reports have indicated that carcinoma cells may be major contributors to the increased circulation of galectins [41], [42]. Furthermore, serum galectins, including galectin-4, have been shown to promote cancer cell adhesion to vascular endothelial cells *in vitro*, suggesting that circulating galectins have a metastasis-promoting effect [40]. These findings may explain the role of venous invasion in metastatic process of lung adenocarcinoma cells.

Alternatively, because metastasis involves several steps and requires the altered expression of many different proteins, other upregulated proteins, including those associated with the glycolysis pathway and poor survival in lung adenocarcinomas [43], may play an important role in cancer metastasis in lung adenocarcinomas with no expression of galectin-4. On the other hand, the number of node-negative carcinoma expressing galectin-4 is relatively high (18.4%) in the present study. One potential explanation for this is that the nodal status was defined at the time of surgery in this study; therefore, we could not evaluate the possibility of the future development of metastasis, including regional LN metastasis after surgery, in the case of adenocarcinomas expressing galectin-4 in the non-metastasized group, in part due to the relatively short follow-up periods in this sample set.

In conclusion, this study describes the possible metastasis-promoting role of galectin-4 in lung adenocarcinoma. Our findings demonstrate that galectin-4 can be a useful biomarker for predicting LN metastasis as well as a potential prognostic factor for patients with lung adenocarcinoma of acinar predominant type.

## Supporting Information

Figure S1 **A representative two-dimensional sodium dodecyl sulfate polyacrylamide gel electrophoresis image of proteins detected in lung adenocarcinoma tissues.** The two spots identified in the regionally metastasized primary tissue sample (P5) were not expressed in the non-metastasized primary tissue sample (N4).
(TIFF)

Figure S2 **Identification of galectin-4 (area square of** **Figure 1** **- P5).** A. The results of Protein Pilot 4.0 search on the data showed human galectin-4 (P56470). Identification of galectin-4 was indicated green and red. There were 31 tryptic peptides found, which corresponded to 59.1% coverage of human galectin-4. B. MS/MS spectrum of *m/z* 531.8 at the [M + 2H] ^2+^ ion (VVFNTLQGGK) with annotated amino acid sequence. C. MS/MS spectrum of *m/z* 824.3 at the [M + 2H] ^2+^ ion (VVVNGNPFYEYGHR) with annotated amino acid sequence. D. MS/MS spectrum of *m/z* 710.8 at the [M + 2H] ^2+^ ion (NSLLNGSWGSEEK) with annotated amino acid sequence.
(TIFF)

Figure S3 **Kaplan-Meier plots for lung adenocarcinomas.** The differences in the overall survival or recurrence-free survival were not significant between galectin-4 positive and galectin-4 negative groups in all lung adenocarcimomas (A, B), lung adenocarcinoma belonging to lepidic predominant type (C, D), lung adenocarcinoma belonging to papillary predominant type (E, F), and lung adenocarcinoma belonging to solid predominant type (G, H).
(TIFF)

Figure S4 **Kaplan-Meier plots for lung adenocarcinomas expressing PPBI.** The differences in the overall survival (A) or recurrence-free (B) survival were not significant between PPBI positive and PPBI negative groups in all lung adenocarcimomas belonging to acinar predominant type.
(TIFF)

Table S1 **Clinicopathological features of discovery samples.**
(PDF)
