# Supplementary figures and images for: Galectin-4, a Novel Predictor for Lymph Node Metastasis in Lung Adenocarcinoma
Source: PLoS One. 2013 Dec 10;8(12):e81883. doi: 10.1371/journal.pone.0081883 (PMC3858289; doi:10.1371/journal.pone.0081883)

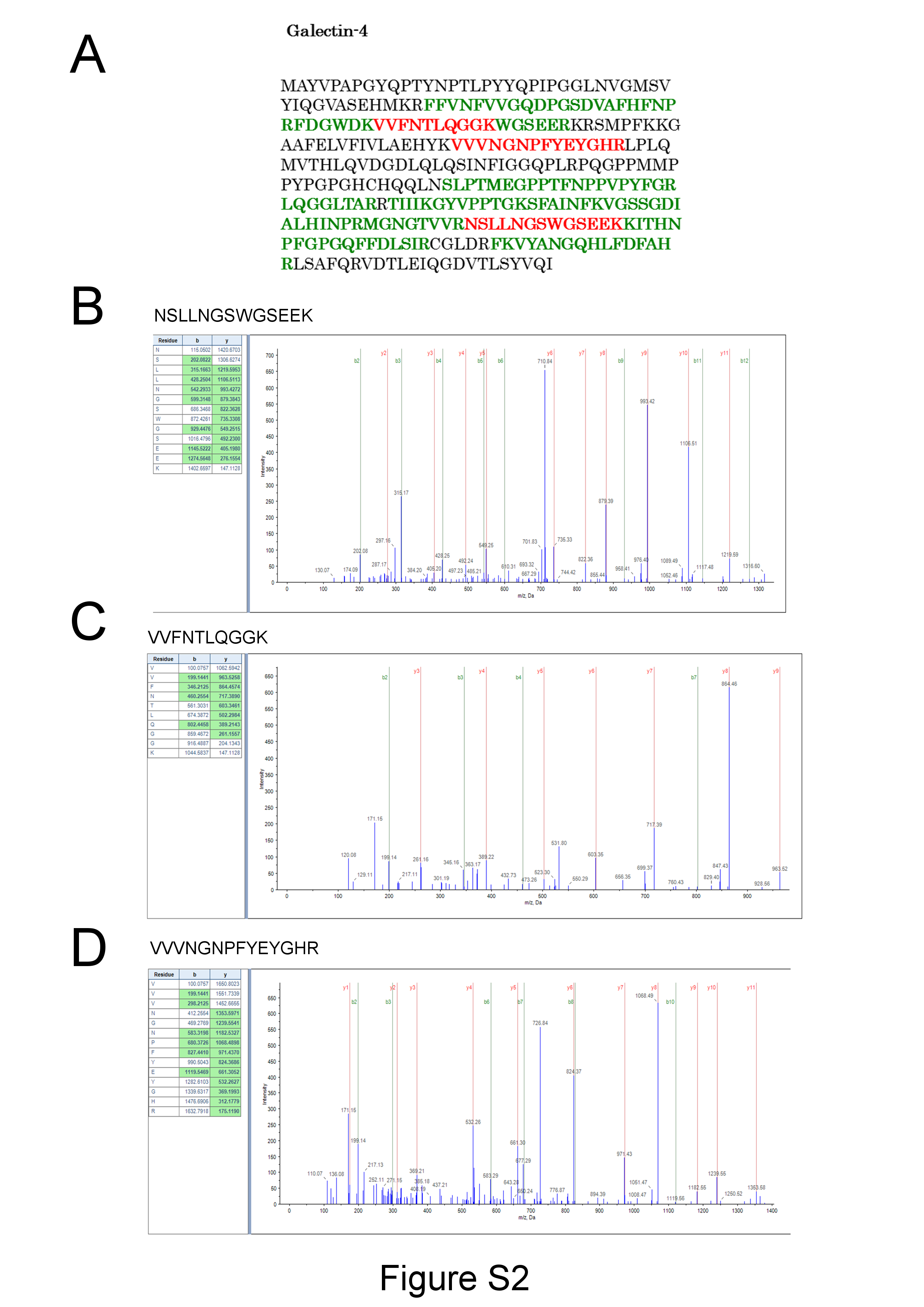

Supplement: Figure S2 — Identification of galectin-4 (area square of Figure 1 - P5). A. The results of Protein Pilot 4.0 search on the data showed human galectin-4 (P56470). Identification of galectin-4 was indicated green and red. There were 31 tryptic peptides found, which corresponded to 59.1% coverage of human galectin-4. B. MS/MS spectrum of m/z 531.8 at the [M + 2H] 2+ ion (VVFNTLQGGK) with annotated amino acid sequence. C. MS/MS spectrum of m/z 824.3 at the [M + 2H] 2+ ion (VVVNGNPFYEYGHR) with annotated amino acid sequence. D. MS/MS spectrum of m/z 710.8 at the [M + 2H] 2+ ion (NSLLNGSWGSEEK) with annotated amino acid sequence. (TIFF) [file pone.0081883.s002.tiff]

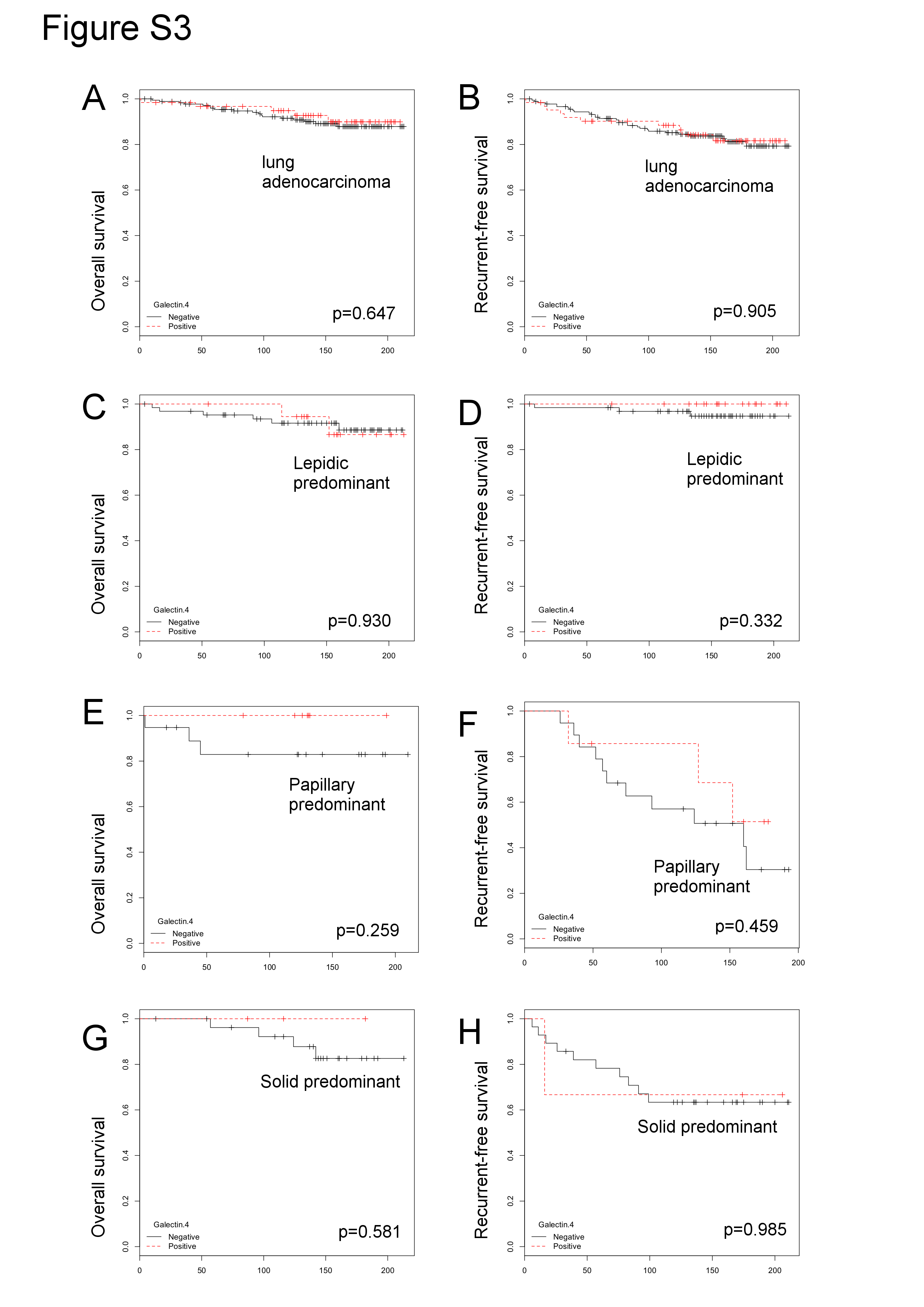

Supplement: Figure S3 — Kaplan-Meier plots for lung adenocarcinomas. The differences in the overall survival or recurrence-free survival were not significant between galectin-4 positive and galectin-4 negative groups in all lung adenocarcimomas (A, B), lung adenocarcinoma belonging to lepidic predominant type (C, D), lung adenocarcinoma belonging to papillary predominant type (E, F), and lung adenocarcinoma belonging to solid predominant type (G, H). (TIFF) [file pone.0081883.s003.tiff]

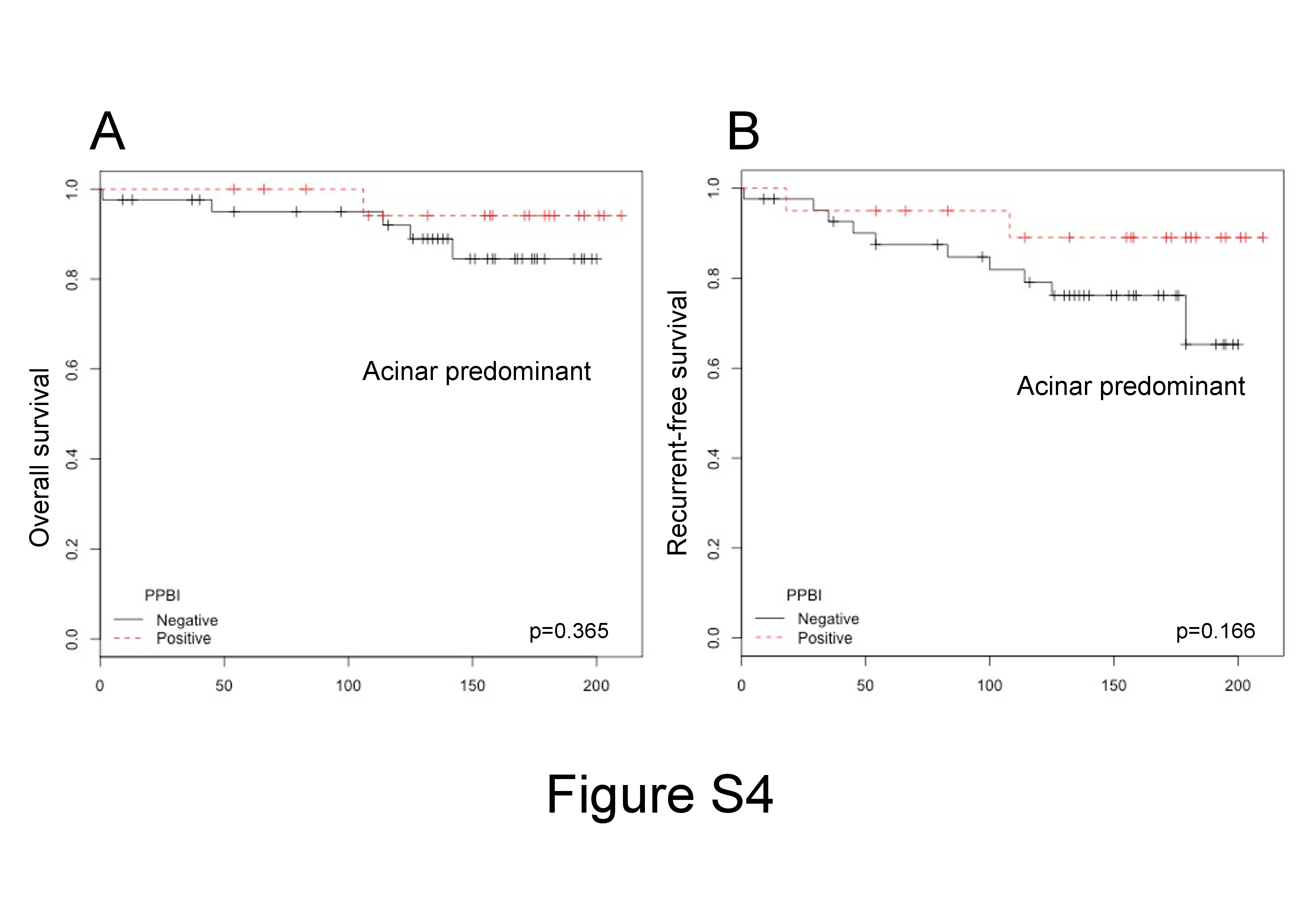

Supplement: Figure S4 — Kaplan-Meier plots for lung adenocarcinomas expressing PPBI. The differences in the overall survival (A) or recurrence-free (B) survival were not significant between PPBI positive and PPBI negative groups in all lung adenocarcimomas belonging to acinar predominant type. (TIFF) [file pone.0081883.s004.tiff]
