# Supplementary material for: Galectin-4, a Novel Predictor for Lymph Node Metastasis in Lung Adenocarcinoma
Source: PLoS One. 2013 Dec 10;8(12):e81883. doi: 10.1371/journal.pone.0081883 (PMC3858289; doi:10.1371/journal.pone.0081883)
Supplement: Table S1 — Clinicopathological features of discovery samples. (PDF) [file pone.0081883.s005.pdf]

Table S1. Clinicopathological features of discovery samples (10 cases)

Sample No	Age	Gender	Histology	pStage
P1	64	M	Solid predominant type	IIA (T1bN1M0)
P2	68	M	Papillary predominant type	IIA (T2aN1M0)
P3	77	M	Papillary predominant type	IIIA (T3aN2M0)
P4	64	M	Papillary predominant type	IIIA (T2aN2M0)
P5	74	F	Papillary predominant type	IIIA (T2aN2M0)
N1	78	M	Acinar predominant type	IA (T1bN0M0)
N2	77	F	Lepidic predominant type	IB (T2aN0M0)
N3	80	M	Lepidic predominant type	IB (T2aN0M0)
N4	71	M	Acinar predominant type	IB (T2aN0M0)
N5	77	F	Acinar predominant type	IIB (T3N0M0)
